# A deep-learning pipeline for the diagnosis and grading of common blinding ophthalmic diseases based on lesion-focused classification model

**DOI:** 10.3389/frai.2024.1444136

**Published:** 2024-09-11

**Authors:** Zhihuan Li, Junxiong Huang, Jingfang Chen, Jin Zeng, Hong Jiang, Lin Ding, TianZi Zhang, Wen Sun, Rong Lu, Qiuli Zhang, Lizhong Liang

**Affiliations:** ^1^Faculty of Medicine, Macau University of Science and Technology, Taipa, Macau, China; ^2^Affiliated Hospital of Guangdong Medical University, Zhanjiang, China; ^3^China Resources Power Intelligent Security Laboratory, Research Institute of Tsinghua University in Shenzhen, Shenzhen, China; ^5^Department of Research and Teaching, The Third People’s Hospital of Shenzhen, Shenzhen, China; ^4^State Key Laboratory of Lunar and Planetary Sciences, Macau University of Science and Technology, Taipa, Macau, China; ^6^The Institute for Sustainable Development, Macau University of Science and Technology, Taipa, Macau, China; ^7^Guangzhou National Laboratory, Guangzhou, China; ^8^Statistical office, Zhuhai People’s Hospital (Zhuhai Clinical Medical College of Jinan University), Zhuhai, China; ^9^Department of Ophthalmology, Affiliated Hospital of Inner Mongolia University for the Nationalities, Tongliao, China; ^10^Department of Ophthalmology Hainan Traditional Chinese Medicine Hospital, Haikou, China; ^11^Yulin First People’s Hospital, Yulin, China; ^12^The Marine Biomedical Research Institute, School of Ocean and Tropical Medicine, Guangdong Medical University, Zhanjiang, Guangdong, China

**Keywords:** artificial intelligence, ensemble learning, deep learning, intelligent diagnosis of multiple fundus diseases, lesion attention

## Abstract

**Background:**

Glaucoma (GLAU), Age-related Macular Degeneration (AMD), Retinal Vein Occlusion (RVO), and Diabetic Retinopathy (DR) are common blinding ophthalmic diseases worldwide.

**Purpose:**

This approach is expected to enhance the early detection and treatment of common blinding ophthalmic diseases, contributing to the reduction of individual and economic burdens associated with these conditions.

**Methods:**

We propose an effective deep-learning pipeline that combine both segmentation model and classification model for diagnosis and grading of four common blinding ophthalmic diseases and normal retinal fundus.

**Results:**

In total, 102,786 fundus images of 75,682 individuals were used for training validation and external validation purposes. We test our model on internal validation data set, the micro Area Under the Receiver Operating Characteristic curve (AUROC) of which reached 0.995. Then, we fine-tuned the diagnosis model to classify each of the four disease into early and late stage, respectively, which achieved AUROCs of 0.597 (GL), 0.877 (AMD), 0.972 (RVO), and 0.961 (DR) respectively. To test the generalization of our model, we conducted two external validation experiments on Neimeng and Guangxi cohort, all of which maintained high accuracy.

**Conclusion:**

Our algorithm demonstrates accurate artificial intelligence diagnosis pipeline for common blinding ophthalmic diseases based on Lesion-Focused fundus that overcomes the low-accuracy of the traditional classification method that based on raw retinal images, which has good generalization ability on diverse cases in different regions.

## Introduction

Despite advancements in medical technology and surgical interventions, the incidence and prevalence of visual impairment remain high globally. In 2020, among the global population of 7.8 billion, it is estimated that 2.2 billion people suffer from some form of visual impairment or blindness, of which at least 1 billion cases could have been prevented or are yet treatable (World Health Organization (WHO), 2016). Visual impairment not only leads to a decreased quality of life but is also associated with a reduction in economic productivity (Wittenborn and Rein, 2013) and an increase in mortality rates (Taylor et al., 2001). The rapid development of deep learning classification algorithms in recent years, with applications in many fields including medical artificial intelligence (AI) (Topol, 2019; Esteva et al., 2019; Norgeot et al., 2019; Ravizza et al., 2019), includes image-based diagnosis (Kermany et al., 2018), voice recognition, and natural language processing (Liang et al., 2019). Particularly, the use of convolutional neural networks with transfer learning assists in efficient and accurate image diagnostics (Kermany et al., 2018; Liang et al., 2019; Wang et al., 2017). In ophthalmology alone, deep learning has been applied for semantic segmentation of retinal images for vessels and optic disc (Zilly et al., 2015), categorizing cataracts (Gao et al., 2014; Long et al., 2017), detecting retinal hemorrhages (Van Grinsven et al., 2016), and interpreting two-dimensional fundus photographs and three-dimensional Optical Coherence Tomography (OCT) images for diagnosing common retinal diseases, including Age-related Macular Degeneration (AMD) (Burlina et al., 2017), Glaucoma (GLAU) (Chen et al., 2015; Li et al., 2018), and Diabetic Retinopathy (DR) (Kermany et al., 2018; Van Grinsven et al., 2016; Burlina et al., 2017; Chen et al., 2015; Gulshan et al., 2016; Ting et al., 2017). Recent studies indicate that AI algorithms can predict cardiovascular risk factors from retinal images, such as age, gender, smoking status, glycated hemoglobin, systolic pressure, and major adverse cardiac events (Poplin et al., 2017), demonstrating that deep learning algorithms can detect subtle correlations not observable to human viewers. Retinal features, such as focal or systemic arteriolar narrowing, arteriovenous nicking, retinal hemorrhages, and retinal nerve fiber layer defects, indicate ocular manifestations of vascular and neuronal diseases. Based on these observations, retinal photographs, which can be acquired rapidly and non-invasively, may serve as “Point of Care” (POC) biomarkers for systemic diseases.

Although artificial intelligence algorithms are developing rapidly, their implementation in the real world poses significant challenges. These include the sourcing of large training datasets, privacy issues surrounding data sharing, the usability and distribution of algorithms, data standardization, the reusability of algorithms across multiple platforms, and the need to meet local regulatory requirements (He et al., 2019). Compared to the developed coastal cities in China, the western regions have relatively scarce medical resources, making the development of an intelligent diagnostic system for blinding ophthalmic diseases, suitable for portable handheld fundus cameras, of great significance. The optical imaging picture quality of portable handheld fundus cameras does not meet the image requirements of medical-specific fundus cameras. Their images suffer from issues such as overexposure, jittery blur, and artifacts. A key challenge is enabling intelligent diagnostic algorithms to avoid these interference areas and make diagnostic decisions. The application of attention mechanisms first gained widespread attention in the field of natural language (Hu, 2018). This mechanism allocates weights as needed, allowing algorithm models to focus more on information-rich areas rather than other areas for decision-making. Integrating attention mechanisms into research for predicting the onset probability of glaucoma and the probability of its late-stage progression based on fundus images is a novel direction (Fei et al., 2022). The fundus anatomical structure and lesion are the diagnostic basis for blinding ophthalmic diseases. Focusing deep learning algorithms on these areas for diagnosis can maximally suppress the interference caused by low-quality fundus images.

Deep learning techniques have been used by many researcher for diagnosis of ophthalmic diseases. However they all used the original fundus images as input to the CNN classification network model for ophthalmic diseases diagnosis (Kermany et al., 2018; Li et al., 2019; Kim et al., 2021). These methods are end-to-end classification tasks, and artifacts, fundus camera imaging difference, etc., may become confusing factors and lead to misdiagnosis. Other researchers (Das et al., 2021) proposed to use semantic segmentation network to first segment retinal vessels, and then input to CNN classification network for DR diagnosis, which is only effective for vascular fundus diseases, and relies heavily on the accuracy of semantic segmentation network. In order to solve the above problems, we propose an intelligent diagnostic algorithm (see Supplementary Figure S5) for common blinding ophthalmic diseases based on deep learning methods.

This algorithm integrates the fundus anatomical structures and lesions into the original fundus images as focal points of attention, the network architecture of which is shown in Supplementary Figure S5. The original RGB fundus image was input into the U-Net semantic segmentation network to obtain the multi-channel binarized lesion image, and then the three-channel lesion fusion map was obtained through a convolutional layer with a convolution kernel size of 3 × 1 × 1 and a fixed weight, in which the convolution kernel corresponding to the red and green channels was 0, and the convolution kernel corresponding to the blue channels was 1. Then, the Threshold activation layer was used to binary the original fundus image, and the “bit-sum” operation was carried out with the Threshold activation layer to obtain the binary blue channel lesion fusion map. The foci fusion image was reversed and the original fundus image was obtained by “bit-sum” operation. Finally, the foci fusion image and the red-green channel image were obtained by “bit-sum” operation, and the foci attention images were classified and learned as inputs of the EfficientNet-B0 convolutional neural network.

**Figure 1:**
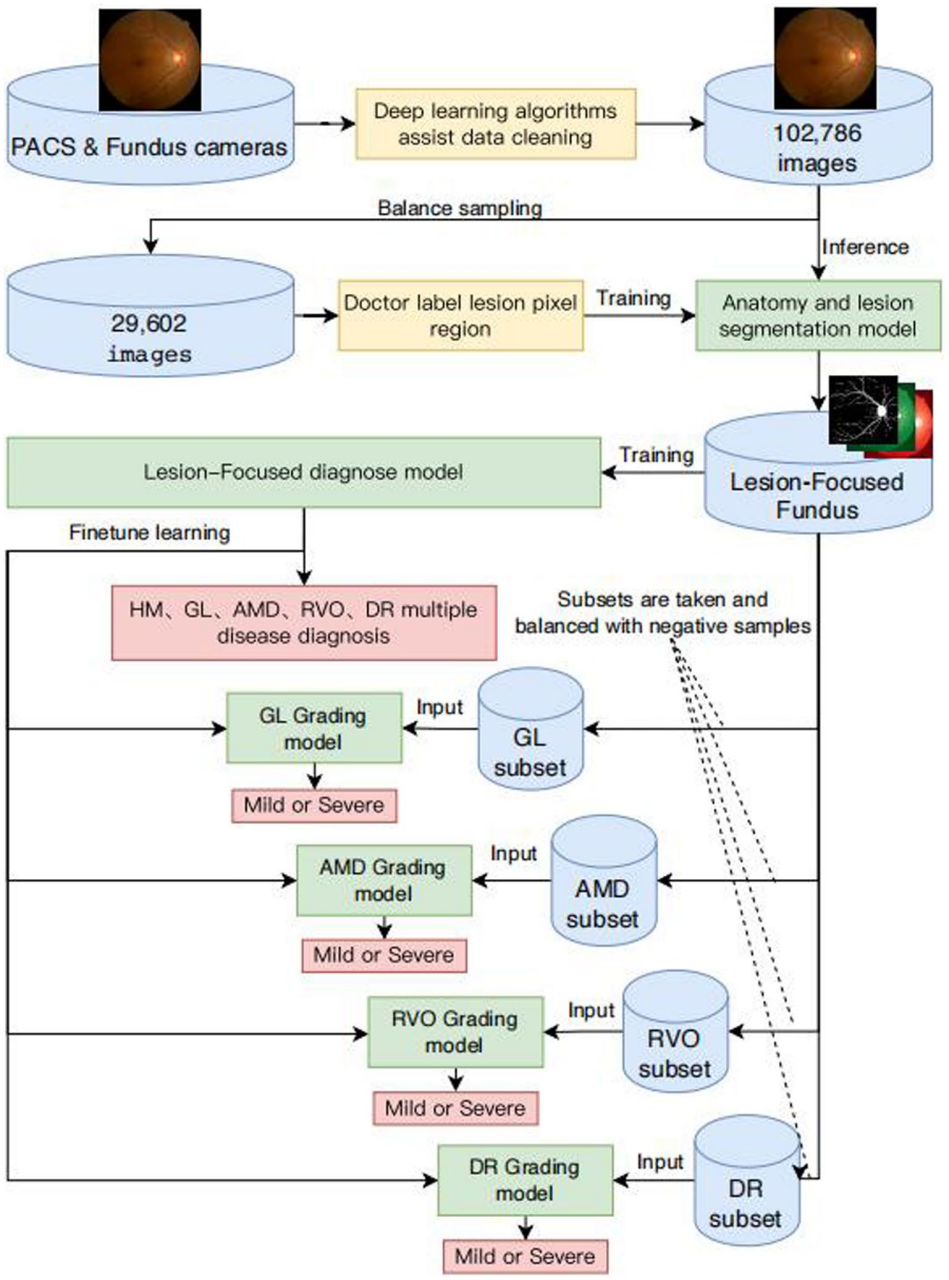
Framework diagram of intelligent diagnosis for common blinding retinal diseases and intelligent grading algorithms for five common blinding retinal diseases.

This article aims to provide healthcare professionals with an effective tool for large-scale retinal disease screening and intelligent diagnostics, facilitating the early detection and timely intervention of common blinding ophthalmic diseases to alleviate individual and economic medical burdens. The framework and flowcharts of our system are shown in Figure 1. We selected common blinding ophthalmic diseases, including Glaucoma (GLAU), Age-related Macular Degeneration (AMD), Retinal Vein Occlusion (RVO), and Diabetic Retinopathy (DR), as they are significant causes of severe visual impairment. The fundus images were sourced from different models of professional and non-professional handheld fundus cameras. We validated the algorithm’s generalizability on an independent patient group dataset of fundus images from Neimeng and Guangxi in China. The algorithm effectively overcame interference from low-quality fundus images and achieved higher accuracy and stronger generalizability compared to traditional deep CNN neural networks.

## Materials and methods

### Data collection and label

We extracted a comprehensive dataset of 102,786 images from retinal examination image databases across various medical institutions. This dataset included color fundus photographs focused on the macula and optic disc, alongside non-fundus images such as reports, blank images, tables, and fundus fluorescein angiography images. We approached the data cleansing process in two stages.

In the first stage, data processors began by selecting a small subset of both non-fundus and fundus color photographs from the initial dataset, exceeding 1 million images, for the inaugural training and validation set of a binary classification algorithm designed for image classification. Subsequently, a different subset of images from the remaining collection was fed into the algorithm for inference. Images misclassified by the algorithm were added to the training set for subsequent rounds of training and validation. This iterative process continued until the entirety of the dataset, comprising over 1 million images, was processed, culminating in the extraction of more than 200,000 fundus images.

During the second stage, we uploaded over 200,000 fundus color photographs to a web-based Computer Vision Annotation Tool (CVAT) system. Five ophthalmologists conducted cross-validation through manual image review to provide medical diagnoses, ensuring each image was reviewed by three ophthalmologists. The consensus diagnosis of two physicians was adopted as the final verdict. Images with divergent diagnoses from all three physicians were randomized and reassigned for review until a definitive diagnosis was established for every image, finalizing the dataset at 102,786 images. Fragments of the data sets are shown in Supplementary Figure S6.

From the refined database of 102,786 fundus images, approximately 5% of images representing each category of blinding ophthalmic diseases were randomly selected to create a dataset for the development of algorithms for semantic segmentation of ocular anatomical structures and lesions. Initially, the dataset was uploaded to the CVAT system for pixel-level independent annotation by five ophthalmologists. Each image received annotations from two physicians, with the intersecting areas of annotations constituting the final standard regions. The identified ocular anatomical structures included the optic cup, optic disc, macula, and blood vessels. The lesions identified encompassed hemorrhage, cotton wool spots, preretinal fibrosis, atrophic crescent, choroidal atrophy, choroidal neovascularization, exudation, macular atrophy, macular degeneration, geographic atrophy, retinal nerve fiber layer defect, retinal defect, scleral show, leopard fundus, retinal neovascularization, vitreous hemorrhage, and retinal detachment.

### Algorithm for encoding multi-channel images of lesions

To minimize input/output (IO) operations and memory consumption during the training of our algorithm, thereby expediting network training, we developed a novel data structure named BITMAP for storing lesion feature maps. This structure comprises 32 channels, divided into groups of 8 channels each. After being encoded in bitmap format, the numerical range of each channel group is [0 ~ 255]. Consequently, these 32 channels can be precisely encoded into PNG image format. For instance, a lesion map with dimensions (512 × 512 × 32), when stored in Numpy format, occupies a file size of 32 M Bytes. In contrast, when encoded as BITMAP and saved as a PNG file, the size is dramatically reduced to 1.8 K Bytes, marking an 18,000-fold reduction in file size. Storing the entire fundus image database in Numpy format would require approximately 6.15 T Bytes of disk space, whereas using BITMAP format reduces this requirement to merely 354 M Bytes. The AI-based diagnostic algorithm in its training phase necessitates numerous iterations of loading the complete fundus database, a process that is highly time-intensive. Consequently, employing Numpy format for this purpose is practically infeasible. Additionally, storing each lesion as an individual grayscale image results in an unwieldy number of files and hampers efficient management. It also necessitates frequent disk accesses during training, thereby hindering the speed of the training process.


(1) $$\left\{ \begin{array}{c} I_{R=}\sum_{\begin{array}{c} i=0,\ldots ,7\\ \; \end{array}}2^{mask_{i}},mask_{i}\in \left\{0,1\right\}\\ I_{G=}\sum_{\begin{array}{c} i=8,\ldots ,15\\ \; \end{array}}2^{mask_{i}},mask_{i}\in \left\{0,1\right\}\\ I_{B=}\sum_{\begin{array}{c} i=16,\ldots ,23\\ \; \end{array}}2^{mask_{i}},mask_{i}\in \left\{0,1\right\}\\ I_{A=}\sum_{\begin{array}{c} i=24,\ldots ,31\\ \; \end{array}}2^{mask_{i}},mask_{i}\in \left\{0,1\right\} \end{array}\right.$$

As illustrated in Equation 1, the process of encoding in BITMAP involves the grayscale images of the Red (R), Green (G), Blue (B), and Alpha (A) channels. Here, denotes the BITMAP layer corresponding to the th channel. In this encoding scheme, points on the foreground object are assigned a value of 1, while all other points are assigned a value of 0. This binary encoding method effectively differentiates foreground objects from the background within each channel of the BITMAP structure.


(2) $$mask_{i}=I^{\left[\frac{i}{8}\right]}2^{i-8\left[\frac{i}{8}\right]},i\in \left[0,31\right]$$

The decoding process of BITMAP, as delineated in Equation 2, involves *mask_i_*, which designates the BITMAP layer for the ith channel. Here, $\left[\frac{i}{8}\right]$ signifies the integer quotient of i divided by 8, and $I_{J}$ (where j∈[0,3]) corresponds to the grayscale images of $I_{R}$, $I_{G}$, $I_{B}$, and $I_{A}$. Our study introduces an innovative storage data structure, BITMAP, for managing multiple lesion data. This structure is constituted of 32 channels of 8-bit grayscale images, shaped as (Height x Width x 32). Each channel records Float32 type values, with 1.0 indicating target pixels and 0.0 indicating background pixels. The 32-channel grayscale images are efficiently compressed and encoded into a 4-channel RGBA image, subsequently stored as a PNG format image. In this encoding, $mask_{0}$ to $mask_{7}$ are transformed into an 8-bit grayscale image for the Red channel of the PNG image, with $mask_{0}$ and $mask_{7}$ corresponding to the low and high bits, respectively. Similarly, $mask_{8}$ to $mask_{15}$, $mask_{16}$ to $mask_{23}$, and $mask_{24}$ to $mask_{31}$ are encoded as 8-bit grayscale images for the Green, Blue, and Alpha channels of the PNG image, respectively, with each mask aligning with either the low or high bits within its channel grouping. This method facilitates efficient storage and retrieval of detailed image data.

### Criteria for diseases diagnostic definition

GLAU: Glaucoma is a group of disorders whose common feature is progressive degeneration of the optic nerve, with loss of retinal ganglion cells, thinning of the retinal nerve fiber layer, and increasing excavation of the optic disc. Intraocular pressure if often the common cause for glaucoma. Glaucoma intraocular pressure is generally above 21 mmHg. It was defined as having two or more of the following features: (1) either vertical expansion of the cup with an optic cup-to-disc ratio greater than or equal to 0.8 or (2) notching manifested as narrowing of the rim tissue at either the superior or inferior rim, together with (3) localized RNFL defects radiating from the optic nerve head. Standard automated perimetry with the Humphrey Field Analyzer was used as an additional diagnostic tool. The perimetric criteria for glaucoma was (pattern standard deviation (PSD) ≥ 5% of normal or glaucoma hemifield test and the mean defect (MD) were outside of normal limits (Li et al., 2004, p. 1594–1595).

AMD: Diagnosing AMD entails distinguishing between macular aging changes and degenerative abnormalities impacting vision. Identifying wet AMD is crucial, as timely intervention can preserve vision. Patients with wet AMD often exhibit specific symptoms alerting to a macular issue beyond general “blurry vision.” (NICE Guideline, No. 82). Our study employs Sigelman’s 1984 four-stage classification for age-related macular degeneration, further categorizing stage I as early and stages II to stages IV as late, based on the presence of exudation within a disc diameter around the macular center.

RVO: Central retinal vein occlusion (RVO) presents as widespread retinal hemorrhages, potentially with optic disc hyperemia or edema, venous dilation, and occluded veins. It encompasses non-ischemic and ischemic types of CRVO and BRVO (Cugati et al., 2006). Ischemic RVO is marked by extensive hemorrhaging and cotton wool spots, with severe vision impairment and late-stage complications like rubeosis and neovascular glaucoma. This study differentiates RVO into mild and severe categories based on clinical severity.

DR: The diagnosis and staging follow the 1984 standards from China’s first National Conference on Fundus Diseases (Li et al., 2004, p. 2169). Stage I features microaneurysms and/or small hemorrhages; Stage II, yellow-white “hard exudates” and/or hemorrhages; Stage III, white “soft exudates” and/or hemorrhages; Stage IV, neovascularization or vitreous hemorrhage on the fundus; Stage V, neovascularization or fibrovascular proliferation; and Stage VI, neovascularization or fibrovascular proliferation with concurrent retinal detachment. In our study, stages I to III are grouped as early, and IV to VI as late, contingent upon the presence of neovascularization.

NORM: Under slit-lamp microscopy, optical sections readily reveal the physiological indentation of the optic nerve head. The internal limiting membrane, seen extending toward the optic nerve head, dips slightly deeper at the physiological cupping. Beneath this, blood vessels are enveloped by a thin tissue layer, revealing fine branches of central vessels. Deeper in the physiological cup, nerve fibers ascend through the lamina cribrosa’s small openings to the papilla’s apex, then curve toward the retina. Optical sections show thinning and indentation of the retina in the macular’s central fovea. The central fovea is characterized by a small, bright spot with a shimmering reflection, positioned right at the forefront of the retinal optical section’s surface (Li et al., 2004, p. 610).

### Feature extraction based on deep learning semantic segmentation network

The U-Net architecture is a symmetrical network structure, comprising three main components: downsampling, upsampling, and skip connections, which give it a shape resembling the letter “U.” The network is bifurcated into a left and a right section. The left part, the Encoder, compresses the image by reducing its dimensions and extracting superficial features through convolution and downsampling processes. On the right, the Decoder enlarges the image size through convolution and upsampling. Convolution in this structure employs valid padding to maintain the integrity of contextual features in the output.

A distinctive feature of the U-Net is its use of skip connections. Each convolutional layer’s feature map is “skip-concatenated” to its corresponding upsampling layer. This design ensures effective utilization of feature maps from each layer in subsequent calculations, thereby integrating both high-level and low-level features. By doing so, U-Net avoids the limitations of performing supervision and loss calculations solely on high-level feature maps. The final feature map in U-net is a composite that includes both high-level and an abundance of low-level features, facilitating the fusion of features across different scales. This unique amalgamation significantly enhances the model’s precision and performance.

In this study, a U-Net semantic segmentation network named Retina-Unet was developed using ResNet18 as its structural foundation, which is depicted in Supplementary Table S3. The features generated by Retina-Unet from fundus images are depicted in Supplementary Figure S4. Initially, RGB color fundus images are resized into a (256 × 256 × 3) matrix. Through convolution operations, the matrix is transformed to (128 × 128 × 64) and then (64 × 64 × 64) following pooling operations. The encoding process involves passing the data through Stage1 to Stage4 Encoders, reshaping it from (64 × 64 × 64) to (8 × 8 × 512). In the decoding phase, the data progresses from Stage0 to Stage4 Decoders, altering the shape from (16 × 16 × 256) to (256 × 256 × 16). The final output is a (256 × 256 × 1) layer, matching the input image size, with the channel count C representing the number of lesion categories to be segmented.

The study acknowledges the morphological diversity among fundus image lesions. Larger lesions like Tessellated retina, Retinal nerve fiber layer defects, and Scleral exposure contrast with smaller ones like Hemorrhage, Cotton woolspot, and Exudation. To address the challenge of accurately classifying both large and small lesions, the research employs separate semantic segmentation networks for each lesion type, ensuring precise segmentation results.

Each fundus anatomical structure and lesion type is analyzed using a dedicated U-Net semantic segmentation network, built upon the ResNet18 backbone. The input layer processes normalized color fundus images. Normalization involves subtracting the RGB matrix of the fundus photo from the average values of a comprehensive fundus image dataset and then dividing by the dataset’s variance, resizing the matrix to 256 × 256. The standard deviation for fundus images is computed by subtracting the mean image from the entire image dataset and calculating the root mean square. This algorithm, trained with float32 numerical values, ensures high precision, retaining 8-decimal accuracy for both mean and standard deviation.

### An intelligent diagnostic algorithm for common blinding ophthalmic diseases based on lesion-focused mechanism

The algorithm for intelligent diagnosis of fundus diseases utilizes the Convolutional Neural Network (CNN), a category of feedforward neural networks characterized by convolutional computations and deep structures, and is one of the cornerstone algorithms in deep learning. Convolutional operations function akin to digital image filters, with each convolutional kernel capable of extracting a singular feature type. To enable the extraction of multiple features, various convolutional kernels can be employed concurrently. An intelligent diagnosis algorithm employing CNN typically comprises convolutional layers, pooling layers, fully connected layers, loss functions, gradient computations, and optimizers.

In this study, the performance of CNN networks is enhanced through the simultaneous enlargement of network width, depth, and resolution, employing the EfficientNet family of networks. As shown in Supplementary Table S4, the network is segmented into nine stages. The initial stage features a standard convolutional layer with a kernel size of and a stride of 2, inclusive of Batch Normalization (BN) and the Swish activation function. Stages two through eight involve the repeated layering of the MBConv structure, with the final column’s “Layers” denoting the repetition count of the MBConv structure within that stage. Stage nine is composed of a standard 1×1 convolutional layer (including BN and Swish activation), an average pooling layer, and a fully connected layer. Each MBConv entry in the table is succeeded by a numeral 1 or 6, indicative of the expansion factor n. This implies that the initial 1×1 convolutional layer in MBConv augments the channels of the input feature matrix by n times, with the symbol or denoting the kernel size used in the Depthwise Conv within MBConv. “Channels” refers to the channels of the output feature matrix post-stage processing (Tan and Le, 2020). Focal attention was given to nine anatomical structures and lesions pertinent to ophthalmic disease diagnosis, including the optic cup, optic disc, macula, and lesions such as hemorrhage, cotton wool spots, preretinal fibrosis, atrophic arc, choroidal atrophy, choroidal neovascularization, exudation, macular atrophy, and leopard fundus. Probability maps of these features were integrated with original fundus images and input into the intelligent diagnosis network, enabling enhanced focus on lesion areas during training and minimizing extraneous information, thereby endowing the neural network with diagnostic capabilities akin to those of a clinical doctor. The training data was segmented into training, internal validation, internal testing, and external validation sets, as delineated in Table 1.

**Table 1:** Summary of fundus image dataset.

Disease	Images of training set	Images of validation set	Images of internal testing set	Images of Neimeng testing set	Images of Guangxi set
GLAU	20,823	3,022	6,003	827	446
AMD	1,594	203	443	988	319
RVO	2,859	411	860	515	429
DR	6,224	832	1,697	1,025	769
NORM	41,108	5,274	11,433	976	2,162

During the training phase, frequent reloading of identical images and computation of lesion-focused fundus images can lead to unnecessary computational overhead and slow down algorithmic training. To address this, caching technology was implemented to encapsulate the generation function of lesion-focused fundus images. Upon its initial invocation, the caching function retrieves the image and lesion image from disk storage, calculates, and returns the lesion-focused image, simultaneously storing the result in the cache. For subsequent invocations with identical parameters, the function retrieves the prior result from the cache, circumventing redundant computations. In terms of computational resource allocation, each algorithm was assigned a Tesla V100 GPU and 30 worker threads for loading fundus images and their corresponding BITMAP-format lesion images.

### An intelligent grading diagnostic algorithm for the five major common blinding ophthalmic diseases based on lesion-focused mechanism

For the development of FiveDisease-Net, an intelligent diagnostic algorithm for five common blinding ophthalmic diseases, subsets of fundus images were selected from the comprehensive Develop-Set. The dataset for algorithm development was divided, based on patient IDs, into training, internal validation, and internal test sets in a 7:1:2 ratio, ensuring no overlap of a single patient’s images across these sets.

The algorithm leverages the structure and pretrained weights of EyeDiagnose-Net as its foundation. It employs fine-tuning training with a fully connected output layer activated by Softmax. This fine-tuning starts from the optimal weights of EyeDiagnose-Net, inheriting previously learned features of fundus images. This strategy accelerates the convergence of the model in new classification tasks, with FiveDisease-Net serving as a base for further training on specific conditions like pathological myopia, glaucoma, age-related macular degeneration, venous occlusion, and diabetic retinopathy. This approach, compared to starting from scratch, results in improved accuracy for the same number of training iterations.

For final diagnostics, the algorithm does not directly use the output results. Instead, it selects a point on the positive output unit’s ROC curve where both sensitivity and specificity are maximized, establishing the final threshold for diagnosis. Output probabilities are categorized as positive or negative based on this threshold. However, the optimal threshold is not always necessary; different thresholds can be applied depending on the diagnostic context. For instance, in large-scale retinal disease screenings, a lower threshold might be used to identify more potential severe cases for timely hospital referral. Conversely, in hospital diagnostic stages, a higher threshold might be chosen to minimize misdiagnosis. This flexible threshold approach tailors the diagnostic process to the specific needs of different medical scenarios.

### Statistics

To evaluate the performance of diagnosis models for disease classification in this study, we calculated confusion matrix and receiver operating characteristic curve (ROC). The models’ performance on binary classification predictions of each disease was evaluated by ROC curves of sensitivity versus 1-specificity. Sensitivity is defined as the proportion of correct model predictions among all results where the true value is Positive. Specificity is defined as the proportion of correctly predicted results by the model among all results where the true value is negative. And accuracy is defined as the proportion of all correctly judged results in the classification model to the total observed values. We find the threshold point on the ROC curve that maximizes the values of sensitivity and specificity, and use this threshold to binary the probability value of each disease output from the diagnosis model into positive and negative categories, and then draw the conflict matrix. Sensitivity and specificity were determined by the selected thresholds on the validation set. We calculated the ratio between the variance of the model outputs and the variance of ground-truthed data using the tuning set to calibrate outputs. We calculated the positive rate for the whole cohort and for each risk group (two strata) as the number of events. The Byar Poisson approximation method was used to calculate 95% CIs of incidence (Breslow, 1978). OR(Odd Ratio) were constructed for different risk groups, and the significance of differences between groups was tested by Chi-square tests.

## Results

### Patient and image characteristics

Our model was trained and validated using a total of 102,786 fundus images from 75,682 individuals, with an average age of 45.5 ± 14.2 years. The training set and internal validation set comprised fundus images captured by professional fundus cameras in Guangdong, China, including 29,848 images of GLAU, 2,240 images of AMD, 4,130 of RVO, 8,753 of DR, and 57,815 of NORM. The fundus images for the two external test sets were sourced from Neimeng and Guangxi, China. All the fundus images were captured using professional fundus cameras. The model was further tested externally with datasets from Neimeng and Guangxi, comprised of professional fundus images: Neimeng’s set had 827 GLAU, 988 AMD, 515 RVO, 1,025 DR, and 976 NORM images, while Guangxi’s included 446 GLAU, 319 AMD, 429 RVO, 769 DR, and 2,162 NORM images, detailed in Table 1.

### Semantic segmentation results of fundus image anatomical structures and fundus lesions

In our study, we independently developed U-Net-based semantic segmentation models, each tailored for one of three fundus anatomical structures or one of fifteen fundus lesions. For each fundus anatomical structure and lesion type, we designed a dedicated binary classification semantic segmentation model, where an output of 1 signifies target detection and 0 represents background. We divided the data into training and validation sets in a 4:1 ratio, ensuring no overlap in patient IDs across these sets (refer to Table 2 for details). The efficacy of these models was assessed using the Intersection over Union (IoU) metric. We achieved commendable segmentation performance for the optic cup, optic disc, and retinal blood vessels - all with IoU scores exceeding 0.77. Similarly, segmentation of larger lesions, such as geographic atrophy, ganglion cell layer defects, and retinal detachment, also yielded positive results, with IoU scores above 0.6. However, the segmentation accuracy for smaller lesions was comparatively lower (detailed in Table 3; Supplementary Figure S7).

**Table 2:** Dataset splitting of training and validation of semantic segmentation of various lesions in fundus images.

	Training		Validation			Training		Validation	
Lesion name	Positive images	Negative images	Positive images	Negative images	Lesion name	Positive images	Negative images	Positive images	Negative images
Disk	1,946	0	482	0	Choroidal neovascularization	350	350	87	87
Cup	1,851	0	461	0	Choroidal atrophy	295	295	73	73
Macular	65	0	14	0	Atrophy arc	339	337	83	80
Vessel	26	0	3	0	Retinal nerve fiber layer defects	400	400	100	100
Hemorrhage	2,070	3,175	517	793	Retinal neovascularization	178	356	44	88
Geographic atrophy	10	20	2	5	Retinal defects	142	142	35	35
Cotton wool spots	800	1,190	200	297	Tessellated retina	263	263	65	65
Exudates	800	2,171	200	541	Sclera exposure	130	130	32	32
Drusen	154	154	38	39	Macular atrophy	214	214	53	53
Vitreous hemorrhage	104	207	25	51	Macular degeration	36	36	9	9

**Table 3:** Anatomical structure and semantic segmentation of fundus images.

Anatomy and lesion name	IoU of training	IoU of validate	Score
Optic cup	0.944777	0.668727	0.779147
Optic disk	0.988255	0.847226	0.903637
Retinal vessel	0.979049	0.847226	0.903637
Macular	0.911657	0.340241	0.568807
Hemorrhage	0.581202	0.298246	0.411429
Cotton-wool spot	0.523801	0.260054	0.365553
Drusen	0.453724	0.212842	0.309194
Atrophic arc	0.423962	0.362179	0.386892
Choroidal atrophy	0.490633	0.280601	0.364614
Choroidal neovascularization	0.477546	0.318491	0.382113
Exudation	0.697246	0.420536	0.531220
Macular degeneration	0.537707	0.085275	0.266248
Geogrraphic atrophy	0.99302	0.350863	0.607726
Retinal nerve fiber layer defects	0.960778	0.651179	0.775019
Retinal defect	0.451425	0.129582	0.258319
Sclera exposure	0.467256	0.197711	0.305529
Tessellation	0.494761	0.37732	0.424297
Neovessels elsewhere	0.996863	0.333022	0.598558
Vitreous hemorrhage	0.604791	0.29893	0.421275
Retinal detachment	0.964422	0.381874	0.614893

### Results of an intelligent diagnostic algorithm focused on lesion-focused for common blinding ophthalmic diseases

**Figure 2:**
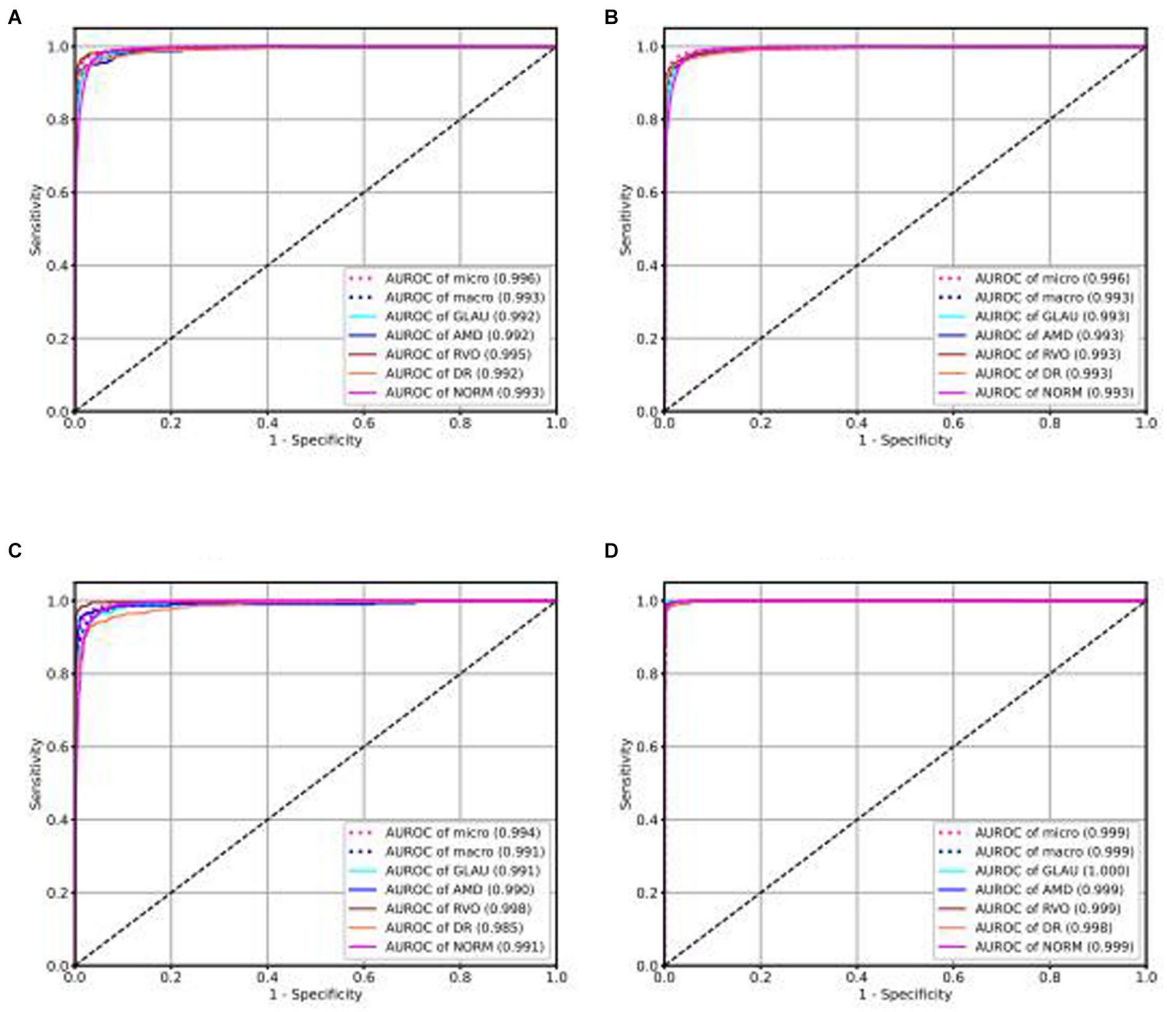
ROC curve of intelligent diagnosis algorithm for common blinding retinal diseases based on the Lesion-Focused model.

Our intelligent diagnostic system can accurately classify common blinding ophthalmic diseases such as GLAU, AMD, RVO, DR, and normal retinal fundus images. It uses a dataset comprising 102,786 fundus images from 75,682 Guangdong patients, referred to as the Develop-Set, for training and validation. We chose the EfficientNet-B0 network, trained from ImageNet (Tan, 2020), as our starting point. After the sixth epoch, no further reduction in validation set loss was observed, so the network weights at this point were saved as the final outcome, resulting in the creation of the EyeDiagnose-Net, an intelligent diagnostic algorithm for the common blinding ophthalmic diseases. The Area Under the Receiver Operating Characteristic Curve (AUROC) was generated to evaluate the model’s ability to distinguish each disease. The internal validation set achieved an micro AUROC of 0.996 and macro AUROC of 0.993, and the AUROC of each blinding retinal disease, from highest to lowest, was RVO (0.995), DR (0.992), AMD (0.992), GLAU (0.992), with normal fundus having a AUROC of 0.993 (see Figure 2A). The internal test set achieved an micro AUROC of 0.996 and macro AUROC of 0.993, and the AUROC of each blinding retinal disease, from highest to lowest, was RVO (0.993), DR (0.993), AMD (0.993), GLAU (0.993), with normal fundus having a AUROC of 0.993 (see Figure 2B).

**Figure 3:**
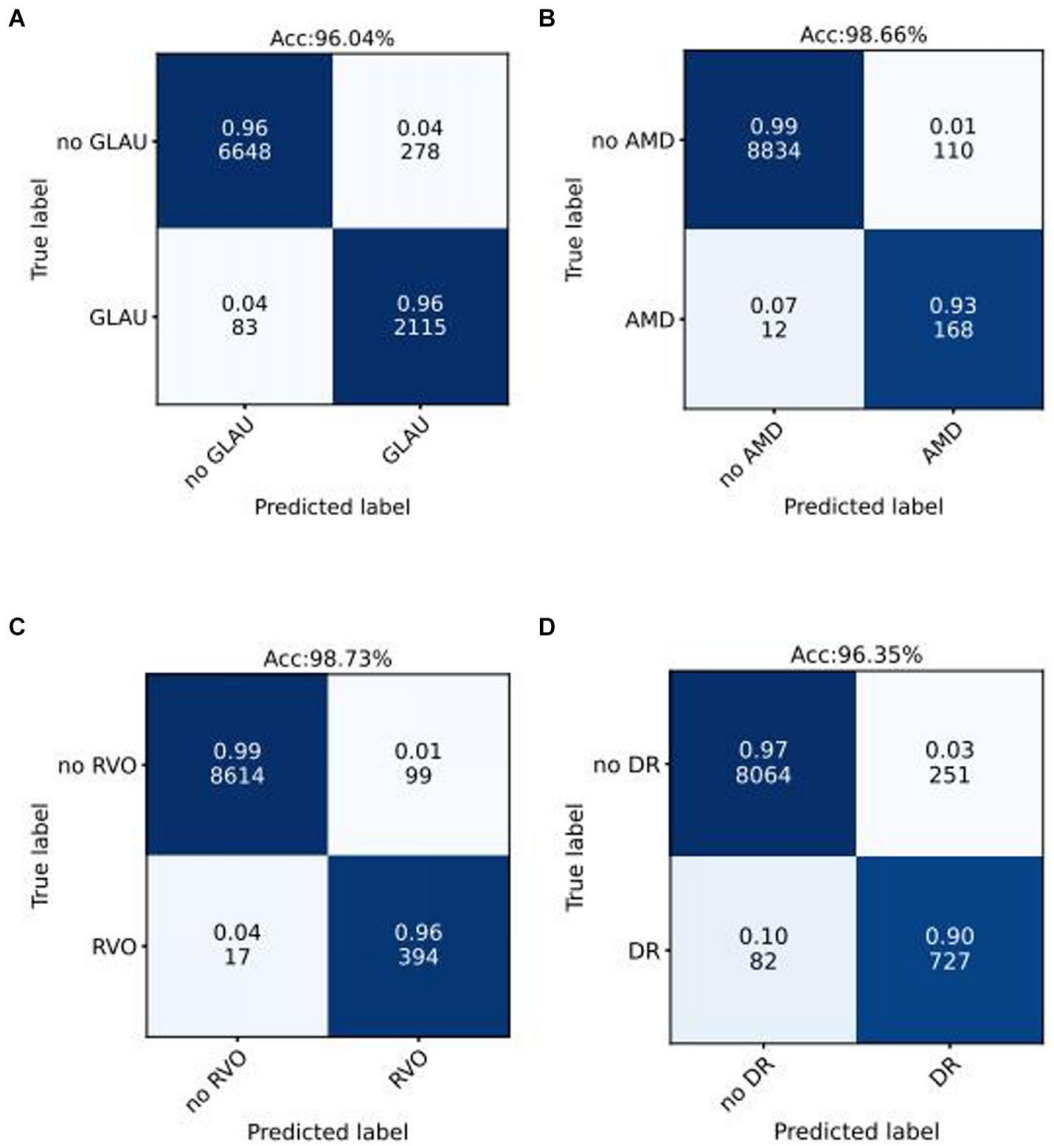
Confusion matrices of internal validation set for common blinding retinal diseases. Acc represents Accuracy, and the decimal above and the integer below in the grid of confusion matrix represents the recall rate and the number of images, respectively.

We use the optimal ROC threshold to distinguish positive and negative samples for each disease. In the internal validation set, as shown in Figures 3A–D, for GLAU, from 2,198 positive samples, 2,115 were correctly identified (sensitivity of 96%), and 278 out of 6,926 negative samples were misclassified as positive, leading to a specificity of 96% and an overall accuracy of 96.04%. For AMD, out of 180 positive samples, 168 were accurately diagnosed (sensitivity of 93%), and 110 out of 8,944 negative samples were incorrectly classified as positive, resulting in a specificity of 99% and an overall accuracy of 98.66%. For RVO, 394 out of 411 positive samples were correctly diagnosed (sensitivity of 96%), and 99 out of 873 negative samples were misclassified as positive, giving a specificity of 99.73% and an overall accuracy of 98.73%. In the assessment of DR, from 727 positive samples, 809 were accurately diagnosed (sensitivity of 90%), and 251 out of 8,315 negative samples were incorrectly classified as positive, leading to a specificity of 97% and an overall accuracy of 96.35%. The confusion matrics of test set were shown in Supplementary Figure S1.

**Figure 4:**
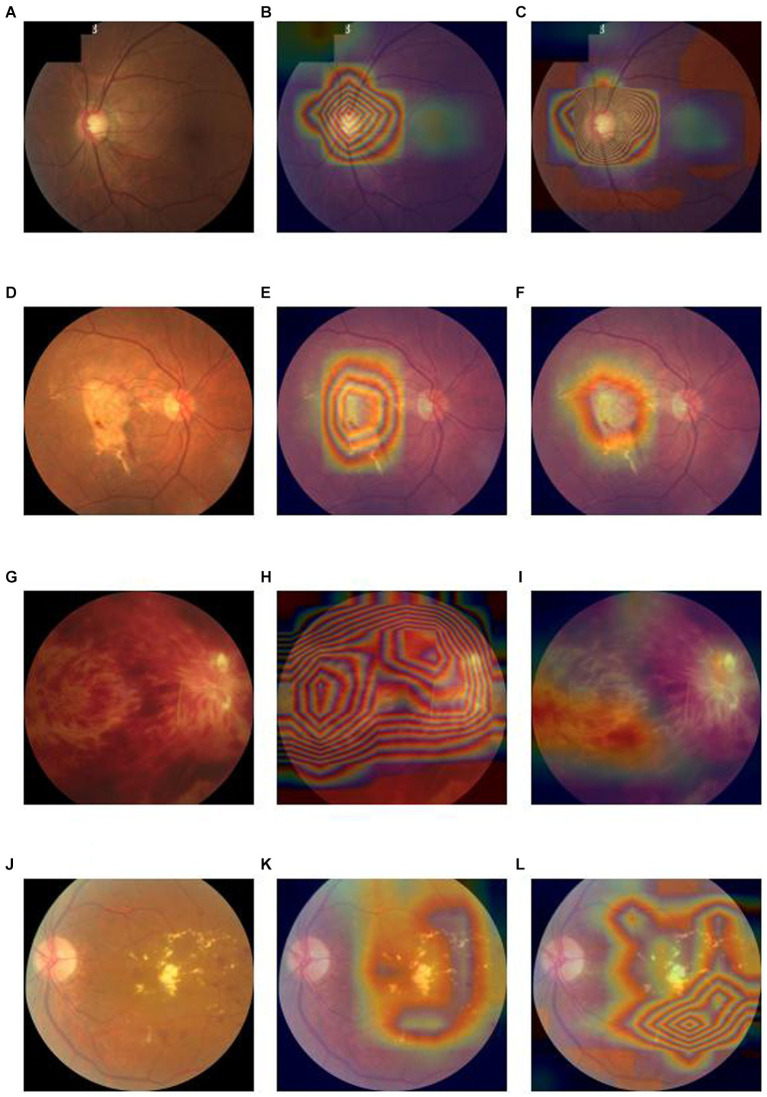
Raw fundus images vs. heatmaps of the Baseline diagnosis model and Lesion-focused diagnosis model.

In terms of interpretability of diagnostic model, our model is also better than the baseline deep convolutional network models. We output the heatmaps of both Lesion-Focused mode and baseline model of the four common blinding eye diseases to find the different evidence. Figure 4 is the heatmaps of the internal validation set, of which Figures 4A–C are the raw fundus image, heatmap of baseline model and heatmap of the Lesion-Focused model of GLAU respectively, the heat is distributed throughout the entire fundus image in Figure 4B, but the heap mainly distribute around the optic cup and macula in Figure 4C, which matches the diagnostic criteria of Glaucoma. Figures 4D–F are the raw fundus image, heatmap of baseline model and heatmap of the Lesion-Focused model of AMD respectively, heap mainly locate in the area of macula, but Figure 4F is more accurate then Figure 4E. Figures 4G–I are the raw fundus image, heatmap of baseline model and heatmap of the Lesion-Focused model of RVO respectively, the distribution of heat appears as contour shape in Figure 4H, but distributed evenly in Figure 4I. Figures 4J–L are the raw fundus image, heatmap of baseline model and heatmap of the Lesion-Focused model of DR respectively, heat also exhibits contour shape distribution in Figure 4K, but evenly distributed in Figure 4L. The results indicate that interpretability of the Lesion-Focused model is more refined and accurate. The heatmaps of internal testing set were shown in Supplementary Figure S2.

### Comparison of performance between traditional models and our lesion-focused models

**Figure 5:**
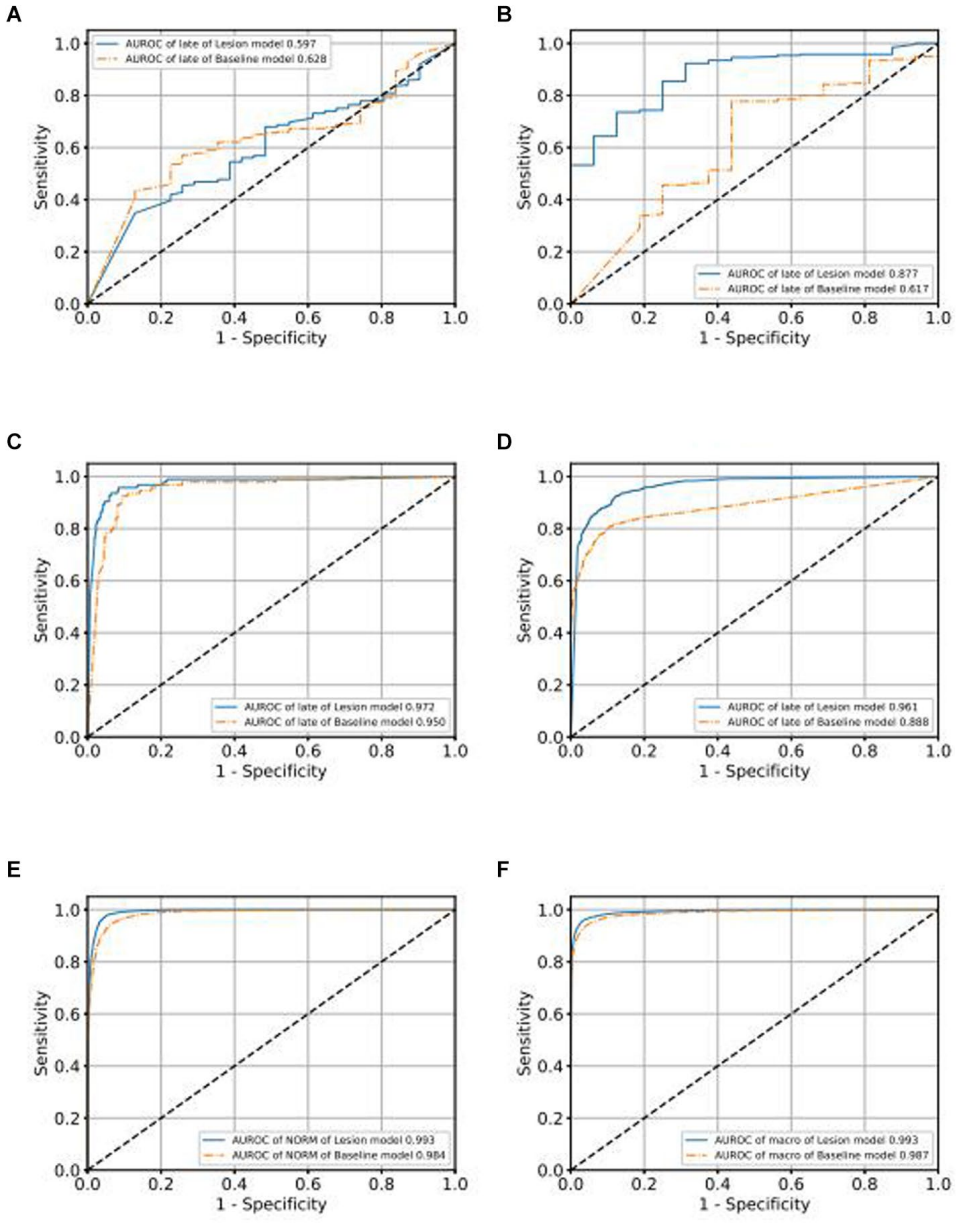
Comparison of ROC curves of Baseline model and Lesion-Focused model in the validation set.

In order to demonstrate the superior performance of our model, we compared the results of our model with those of traditional classification models. As depicted in Figure 5 for the validation set, we observe that categories, Figures 5A–F represent the Receiver Operating Characteristic (ROC) curves for classifications of various conditions, including GLAU, AMD, RVO, DR, NORM, and macro. Our model’s AUROC consistently surpasses 0.99 across these conditions. Apart from the classification of RVO, it outperforms the baseline model in all cases. Moreover, in the lesions where our model leads, our ROC curve is always higher than the baseline ROC curve, indicating that our model’s results are superior to the baseline at various thresholds. This finding extends to the test set as well, where Supplementary Figures S3A–F likewise denote the ROC curves for the aforementioned conditions. Here, our model maintains an AUROC value around 0.993, demonstrating consistency with the validation set and indicating satisfactory generalization capabilities. In the test set scenario, our model’s AUROC values for all conditions exceed those of the baseline, with our ROC curves similarly outstripping the baseline’s across all evaluated conditions.

### Our lesion-focused diagnostic model is a key factor of the diseases

Additionally, to assess the classification efficacy of our intelligent diagnostic model, we employed the Odds Ratio (OR) to evaluate the impact of specific factors on the occurrence rates of particular events at designated time points. The median of the risk scores, derived from the diagnostic model, was utilized to categorize the fundus images in the internal validation set into high and low-risk groups.

The ORs were reported alongside 95% Confidence Intervals (CIs), calculated using the Byar Poisson approximation method (Breslow, 1978).

Table 4 illustrates that, for the four principal common blinding ophthalmic diseases, the Positive events in both high and low scoring groups denote the count of positive sample images. The positive rates were calculated as the proportion of positive samples in relation to the total sample count within each group. These Positive rates were also reported with 95% CIs, estimated via the non-parametric bootstrap method, involving 1,000 random resamplings with replacement.

**Table 4:** Predicted positive rates of common blinding ophthalmic diseases for the validation set, stratified by risk level.

Subset	No. of patient (95% CI)	Age(mean and std years)	Gender(Male/Female)	Positive events	Positive rate(95% CI)	OR(95% CI)	*p*-value
Prognostic analysis: GL							
Low risk	4,983	23.6(15.7)	2564/2419	160	0.07(0.06, 0.08)	NA	NA
High risk	1,655	39.6(12.3)	816/839	2,290	0.93(0.9, 0.97)	605.8(474.8, 778.8)	<0.001
Prognostic analysis: AMD							
Low risk	6,251	27.6(16.3)	3170/3081	11	0.06(0.03, 0.11)	NA	NA
High risk	336	37.9(16.3)	180/156	169	0.94(0.8, 1.0)	656.2(363.3, 1165.7)	<0.001
Prognostic analysis: RVO							
Low risk	6,072	26.3(15.3)	3079/2993	9	0.02(0.01, 0.04)	NA	NA
High risk	556	49.7(14.7)	300/256	402	0.98(0.88, 1.0)	1456.2(777.9, 2534.6)	<0.001
Prognostic analysis: DR							
Low risk	5,888	26.6(16.1)	3008/2880	78	0.1(0.08, 0.12)	NA	NA
High risk	720	39.6(15.1)	362/358	731	0.9(0.84, 0.97)	279.4(220.9, 350.9)	<0.001

A chi-square test was applied for hypothesis testing across the nine major common blinding ophthalmic diseases. The findings indicated a significant disparity between the high and low-risk groups for each disease (*p* < 0.001), thereby affirming the reliable discriminatory capacity of the intelligent diagnostic model for each type of blinding retinal disease. Further details are provided in Table 4.

To gauge the diagnostic algorithm’s generalizability, analogous testing was performed on both the internal and external test sets. Consistently, the high-risk group for each common blinding retinal disease shown a significant difference from the low-risk group (*p* < 0.001), as detailed in Supplementary Tables S1, S2.

### Artificial intelligent algorithms of grading of fundus of common blinding ophthalmic diseases

Staging of fundus diseases, based on the characteristics and severity of retinal lesions, is pivotal for timely disease detection and treatment. This study extends its focus to intelligent grading diagnosis methodologies for four prevalent fundus diseases: Glaucoma (GLAU), Age-related Macular Degeneration (AMD), Retinal Vein Occlusion (RVO), and Diabetic Retinopathy (DR). We fine-tune the pretrained EyeDiagnose-Net to develop grading algorithms for these four major blinding retinal conditions.

**Figure 6:**
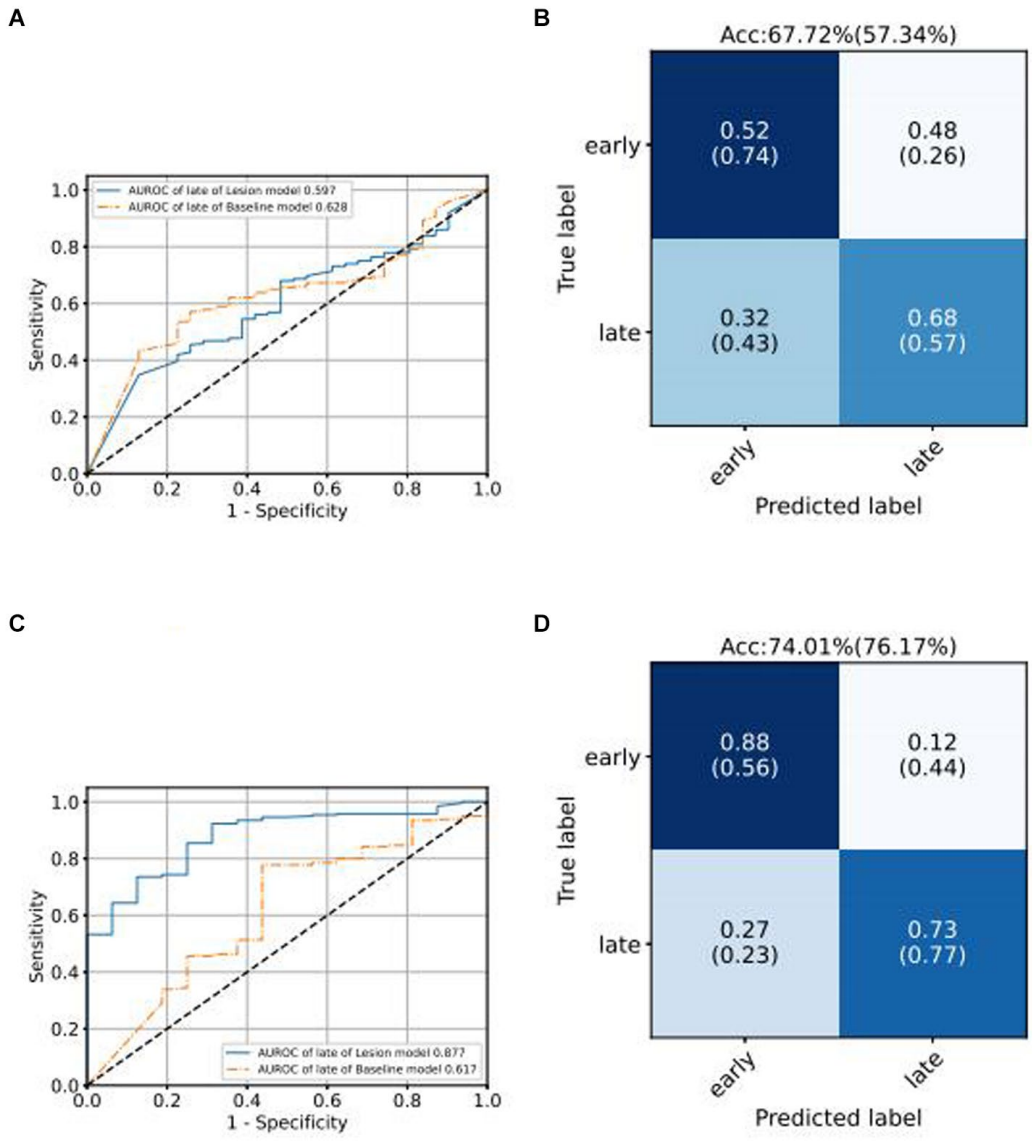
Comparison of ROC curves of Baseline model and Lesion-Focused model in the test set.

**Figure 7:**
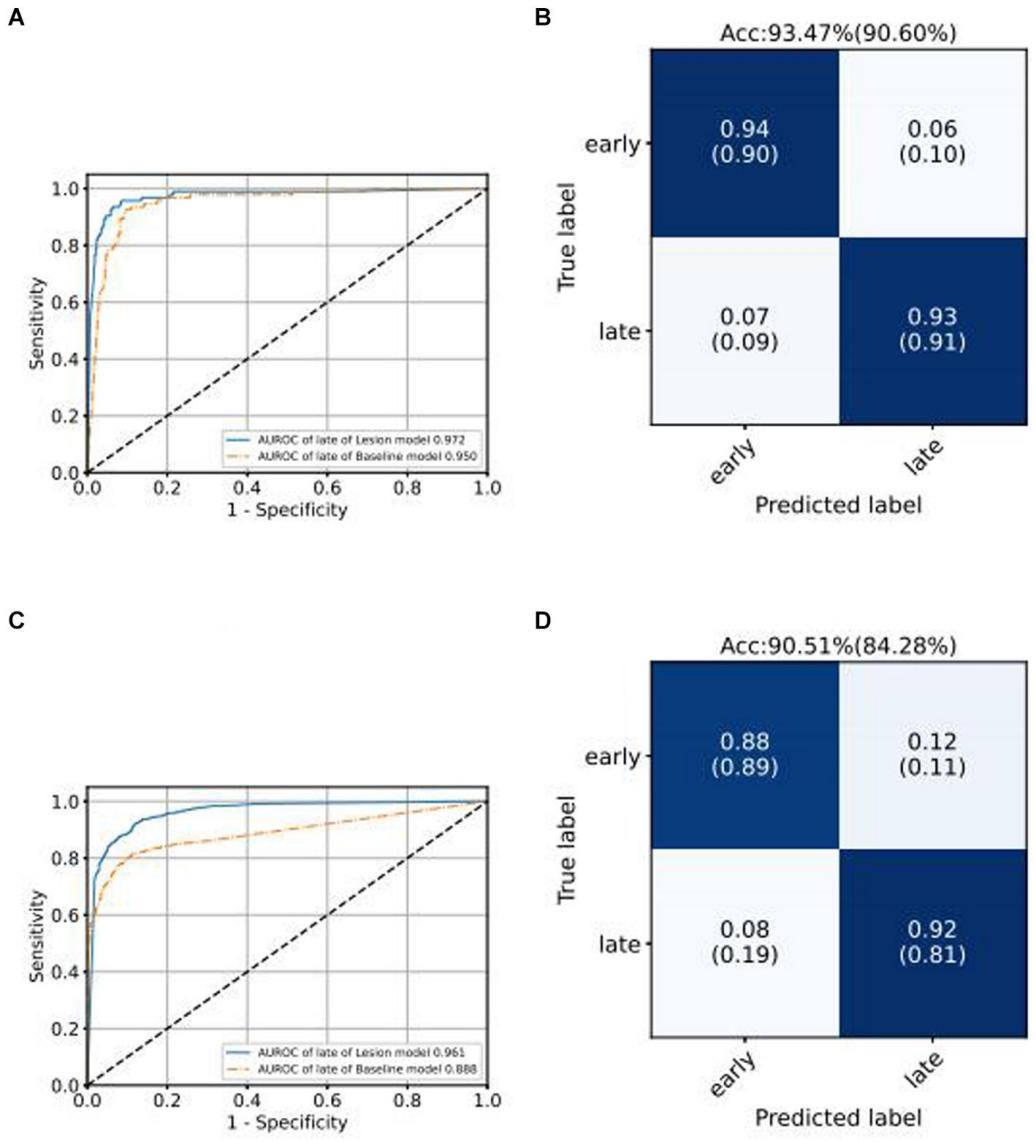
Comparison of ROC curves of Baseline model and Lesion-Focused model in the test set.

We use the ROC curve to measure the performance of the grading algorithms model, and then find the best threshold on the ROC curve to distinguish early and late samples, maximizing specificity and sensitivity, and calculating the confusion matrix. Figures 6, 7 present the internal validation results of the intelligent grading algorithms for these major blinding ophthalmic diseases. From the AUROC metric, our model performs significantly better than the Baseline model in AMD, RVO, and DR diseases, with AMD 0.877 vs. 0.617, RVO 0.972 vs. 0.950, and DR 0.961 vs. 0.888, respectively, shown in Figures 6C–D, 7A–D. Our model does not perform as well as the Baseline model in terms of AUROC for the GLAU disease, but overall accuracy and the recall of late stage are 10 percentage points higher, with 67.72% vs. 57.34% and 0.68 vs. 0.57, respectively, shown in Figures 7A,B.

### Model performance in independent external testing set

We conducted external validation of our model using independent groups from distinct geographic regions: Neimeng and Guangxi of China, detailed in Figures 2C,D. The dataset for Neimeng included 4,125 fundus images, whereas the Guangxi dataset comprised 4,331 fundus images. Our intelligent diagnostic model demonstrated high accuracy across all four evaluated common blinding ophthalmic diseases, illustrating its widespread applicability to diverse global data sets. For the Neimeng dataset, the AUROC of the intelligent diagnostic algorithm for each disease surpassed 0.994, as depicted in Figure 2C, of which the AUROCs are GLAU (0.991), AMD (0.990), RVO (0.998), DR (0.985), and NORM (0.991). In the Guangxi dataset, the AUROC for the diseases ranged as follows: GLAU (0.991), AMD (0.990), RVO (0.998), DR (0.985), and NORM (0.991), shown in Figure 2D. The algorithm’s performance in intelligent diagnosis was marginally better in the Guangxi group compared to the Neimeng group.

## Discussion

For individuals exhibiting early-stage fundus symptoms, artificial intelligence (AI) screening algorithms serve as efficient tools for mass screening, enabling prompt detection of disease indicators and facilitating access to medical care. This approach is key in managing the onset and spread of diseases. Additionally, AI predictive algorithms are valuable for mass screening in asymptomatic populations, predicting the likelihood of disease development in the future based on normal fundus images without lesions. This predictive capability aids in early identification of potential patients, allowing for early diagnosis and intervention.

This paper presents an innovative algorithm, the Lesion-Focused Ophthalmic Disease Intelligent Diagnostic Algorithm, which combines semantic segmentation and classification techniques. This algorithm stands out from traditional neural network approaches by offering greater interpretability and faster convergence. The integration of medical expertise in the neural network training, particularly through the semantic segmentation of anatomical structures and lesions, renders the diagnostic outcomes more clinically relevant. Furthermore, building on the intelligent diagnostic algorithm for common blinding ophthalmic diseases, we developed a specialized intelligent staging diagnostic algorithm for five major blinding ophthalmic diseases. This was achieved by fine-tuning training with a minimal dataset. The effectiveness of this algorithm is on par with medical professionals, attributed to the incorporation of medical expertise into the training process via attention mechanisms.

The effectiveness of semantic segmentation networks is linked to the diversity of training data. In this study, to maximize learning from various sample types, 500 fundus images for each disease were randomly selected from a comprehensive dataset and annotated by five ophthalmologists for anatomical structures and lesion areas. This task was challenging due to the subtlety of lesions, and imprecise annotations could greatly affect network accuracy. To mitigate these issues, the online CVAT annotation tool was employed, enabling real-time annotation updates by doctors.

Our proposed intelligent diagnostic algorithm demonstrated superior performance over a conventional algorithm that used original fundus images for training. This superiority was evident in faster convergence during training and more stable accuracy in validation sets. The new algorithm achieved optimal performance by the second training round, surpassing the traditional approach based on original fundus images. This improvement is attributed to the significant role of anatomical structures and lesion areas in the training process, focusing the network’s attention on critical areas. The lesion attention mechanism not only boosted the network’s interpretability but also enabled it to diagnose similarly to a clinical doctor. Detail information is shown in Supplementary Figure S8.

In screening for blinding ophthalmic diseases, higher disease sensitivity is crucial to avoid missed diagnoses. Our study innovatively trained a multi-label classification task using single-label annotated data, leading to a sophisticated ophthalmic disease screening algorithm. This algorithm can deduce combinations of nine different diseases from a single fundus image input, demonstrating high sensitivity and specificity, often exceeding 0.90 across various test sets including internal, external tests in diverse populations (Neimeng and Guangxi). When comparing the original fundus baseline model with the Lesion-Focused model, the latter consistently shown superior performance in accuracy, sensitivity, and specificity across all test sets (Supplementary Figure S3), underscoring the enhanced screening efficacy of the Lesion-Focused approach.

In the field of neural network training, starting with a smaller dataset typically leads to suboptimal outcomes due to the initial random iteration of weights and slow learning progression. However, initiating training from pre-trained weights enables the network to possess baseline recognition capabilities, allowing it to quickly reach satisfactory performance levels after minimal fine-tuning with new data. In our approach, we first developed an intelligent diagnostic network for five major blinding fundus diseases. We then modified the final output layer from a six-category to a two-category classification, creating a staging diagnostic model. This model was further enhanced through transfer learning to establish intelligent diagnostic staging networks for specific diseases, including GLAU, AMD, RVO, and DR staging diagnostics.

From a clinical perspective, diagnosing common blinding ophthalmic diseases typically requires a holistic analysis of various examination results, such as clinical assessments, optic nerve head imaging, and visual field tests. Our study, however, relied solely on fundus images due to their high accessibility and usability. Future studies may look into integrating additional data modalities to increase the predictive algorithms’ accuracy. A limitation of our current approach is the exclusive use of high-quality fundus images, which restricts applicability in cases with medium opacity that hinder the clarity of such images. Additionally, the relatively low incidence of the nine major common blinding ophthalmic diseases in the general population resulted in a limited number of cases for our study. Enhancing the predictive model’s accuracy in the future could involve incorporating a more extensive set of training data.

## Data availability statement

The raw data supporting the conclusions of this article will be made available by the authors, without undue reservation.

## Ethics statement

The studies involving humans were approved by the Clinical Research Ethics Committee of the Affiliated Hospital of Guangdong Medical University. The studies were conducted in accordance with the local legislation and institutional requirements. The participants provided their written informed consent to participate in this study.

## Author contributions

ZL: Writing – original draft, Writing – review & editing. JH: Writing – review & editing. JC: Data curation, Writing – review & editing. JZ: Data curation, Writing – review & editing. HJ: Formal analysis, Writing – review & editing. LD: Data curation, Writing – review & editing. TZ: Formal analysis, Writing – review & editing. WS: Methodology, Writing – review & editing. RL: Writing – review & editing. QZ: Supervision, Writing – review & editing. LL: Funding acquisition, Supervision, Writing – review & editing.
