# Supplementary material for: A deep-learning pipeline for the diagnosis and grading of common blinding ophthalmic diseases based on lesion-focused classification model
Source: Front Artif Intell. 2024 Sep 11;7:1444136. doi: 10.3389/frai.2024.1444136 (PMC11422385; doi:10.3389/frai.2024.1444136)
Supplement: Supplementary file 1 [file Table_1.docx]

Table S1: Predicted positive rates of common blinding ophthalmic diseases for the Internal test set, stratified by risk level.

| **Subset** | **Num of patient(95% CI)** | **Age (mean and std years)** | **Gender (Male/Female)** | **Positive events** | **Positive rate(95% CI)** | **OR(95% CI)** | **P value** |
| --- | --- | --- | --- | --- | --- | --- | --- |
| **Prognostic analysis: GL** | | | | | | | |
| Low risk | 10389 | 23.4(15.7) | 5281/5108 | 345 | 0.07(0.06,0.08) | NA | NA |
| High risk | 3353 | 39.2(12.6) | 1690/1663 | 4464 | 0.93(0.9,0.96) | 592.8(505.4,705.2) | <0.001 |
| **Prognostic analysis: AMD** | | | | | | | |
| Low risk | 12949 | 27.1(16.3) | 6567/6382 | 27 | 0.07(0.04,0.1) | NA | NA |
| High risk | 717 | 38.9(16.2) | 376/341 | 378 | 0.93(0.84,1.0) | 560.4(395.7,784.6) | <0.001 |
| **Prognostic analysis: RVO** | | | | | | | |
| Low risk | 12607 | 25.8(15.3) | 6365/6242 | 41 | 0.05(0.03,0.06) | NA | NA |
| High risk | 1065 | 50.1(14.5) | 586/479 | 819 | 0.95(0.89,1.0) | 629.4(475.9,826.1) | <0.001 |
| **Prognostic analysis: DR** | | | | | | | |
| Low risk | 12251 | 26.1(15.9) | 6226/6025 | 165 | 0.1(0.09,0.12) | NA | NA |
| High risk | 1450 | 40.4(15.9) | 724/726 | 1449 | 0.9(0.85,0.95) | 250.5(214.6,292.5) | <0.001 |

Table S2: Predicted positive rates of common blinding ophthalmic diseases for the Neimeng and Guangxi test external set, stratified by risk level.

| **Subset** | **Num of patient(95% CI)** | **Age (mean and std years)** | **Gender (Male/Female)** | **Positive events** | **Positive rate(95% CI)** | **OR(95% CI)** | **P value** |
| --- | --- | --- | --- | --- | --- | --- | --- |
| **Prognostic analysis: GL** | | | | | | | |
| Low risk | 1793 | 24.2(15.4) | 958/835 | 37 | 0.03(0.02,0.04) | NA | NA |
| High risk | 583 | 33.5(15.2) | 304/279 | 1336 | 0.97(0.92,1.0) | 1458.5(851.1,2659.5) | <0.001 |
| **Prognostic analysis: AMD** | | | | | | | |
| Low risk | 2086 | 25.0(15.2) | 1102/984 | 24 | 0.02(0.01,0.03) | NA | NA |
| High risk | 190 | 42.1(15.1) | 105/85 | 1332 | 0.98(0.93,1.0) | 3232.1(2189.7,4644.5) | <0.001 |
| **Prognostic analysis: RVO** | | | | | | | |
| Low risk | 2200 | 25.7(15.4) | 1174/1026 | 22 | 0.02(0.01,0.03) | NA | NA |
| High risk | 65 | 51.1(14.8) | 31/34 | 941 | 0.98(0.92,1.0) | 4723.9(3118.7,6971.0) | <0.001 |
| **Prognostic analysis: DR** | | | | | | | |
| Low risk | 1831 | 23.2(14.4) | 979/852 | 37 | 0.02(0.02,0.03) | NA | NA |
| High risk | 515 | 36.6(16.2) | 269/246 | 1678 | 0.98(0.93,1.0) | 691.2(513.9,916.2) | <0.001 |

Table S3: U-Net network structure

| Stage | Operator | Resolution | Channels |
| --- | --- | --- | --- |
| 1 | input_data | 256x256 | 3 |
| 2 | conv0 | 128x128 | 64 |
| 3 | pooling0 | 64x64 | 64 |
| 4 | encoder_stage1 | 64x64 | 64 |
| 5 | encoder_stage2 | 32x32 | 128 |
| 6 | encoder_stage3 | 16x16 | 256 |
| 7 | encoder_stage4 | 8x8 | 512 |
| 8 | decoder_stage0 | 16x16 | 256 |
| 9 | decoder_stage1 | 32x32 | 128 |
| 10 | decoder_stage2 | 64x64 | 64 |
| 11 | decoder_stage3 | 128x128 | 32 |
| 12 | decoder_stage4 | 256x256 | 16 |
| 13 | output | 256x256 | C |

Table S4: EfficientNet-B0 network structure

| Stage | Operator | Resolution | Channels | Layers |
| --- | --- | --- | --- | --- |
| 1 | Conv3x3 | 224x224 | 32 | 1 |
| 2 | MBConv1,k3x3 | 112x112 | 16 | 1 |
| 3 | MBConv6,k3x3 | 112x112 | 24 | 2 |
| 4 | MBConv6,k5x5 | 56x56 | 40 | 2 |
| 5 | MBConv6,k3x3 | 28x28 | 80 | 3 |
| 6 | MBConv6,k5x5 | 14x14 | 112 | 3 |
| 7 | MBConv6,k5x5 | 14x14 | 192 | 4 |
| 8 | MBConv6,k3x3 | 7x7 | 320 | 1 |
| 9 | Conv1x1 & Pooling & FC | 7x7 | 1280 | 1 |
