# Supplementary material for: A deep-learning pipeline for the diagnosis and grading of common blinding ophthalmic diseases based on lesion-focused classification model
Source: Front Artif Intell. 2024 Sep 11;7:1444136. doi: 10.3389/frai.2024.1444136 (PMC11422385; doi:10.3389/frai.2024.1444136)
Supplement: Supplementary file 2 [file Data_Sheet_1.PDF]

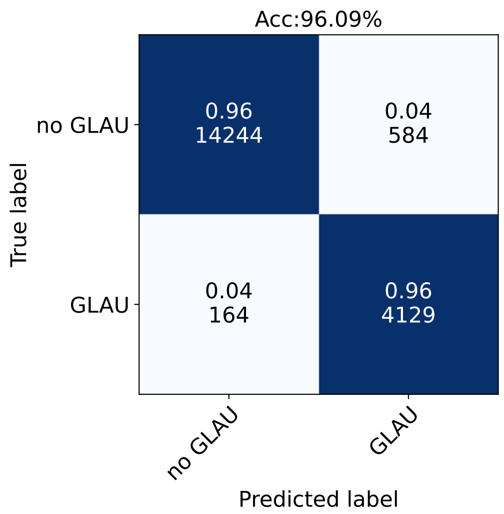

(a)GLAU

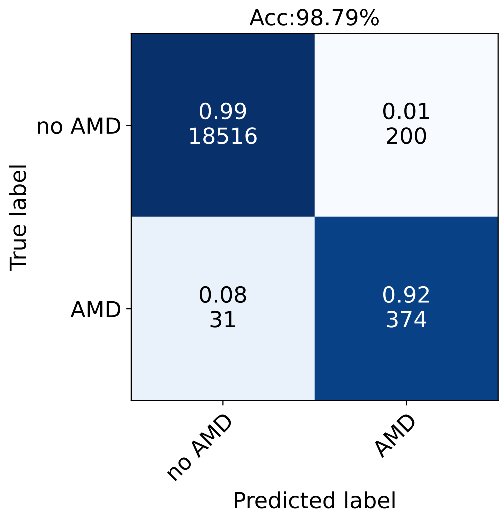

(b)AMD

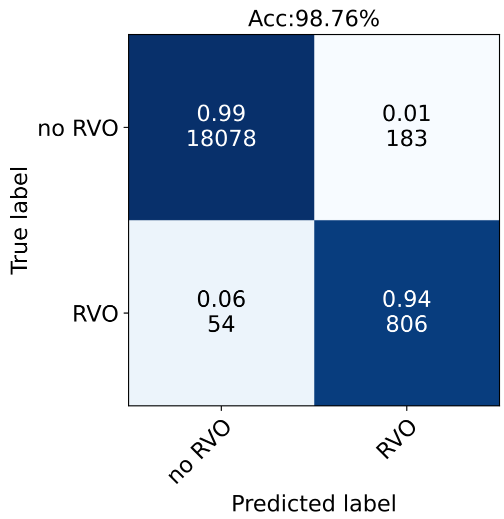

(c)RVO

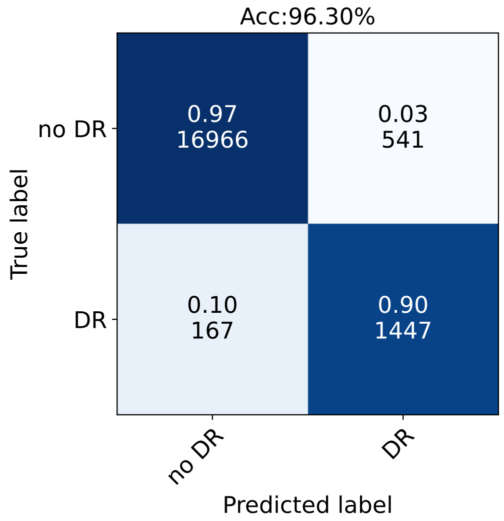

(d)DR

Figure S1: Confusion matrices of internal test sets for the common blinding retinal diseases. Acc represents Accuracy, and the decimal above and the integer below in the grid of confusion matrix represents the recall rate and the number of images respectively.
