# Supplementary material for: A deep-learning pipeline for the diagnosis and grading of common blinding ophthalmic diseases based on lesion-focused classification model
Source: Front Artif Intell. 2024 Sep 11;7:1444136. doi: 10.3389/frai.2024.1444136 (PMC11422385; doi:10.3389/frai.2024.1444136)
Supplement: Supplementary file 4 [file Data_Sheet_3.PDF]

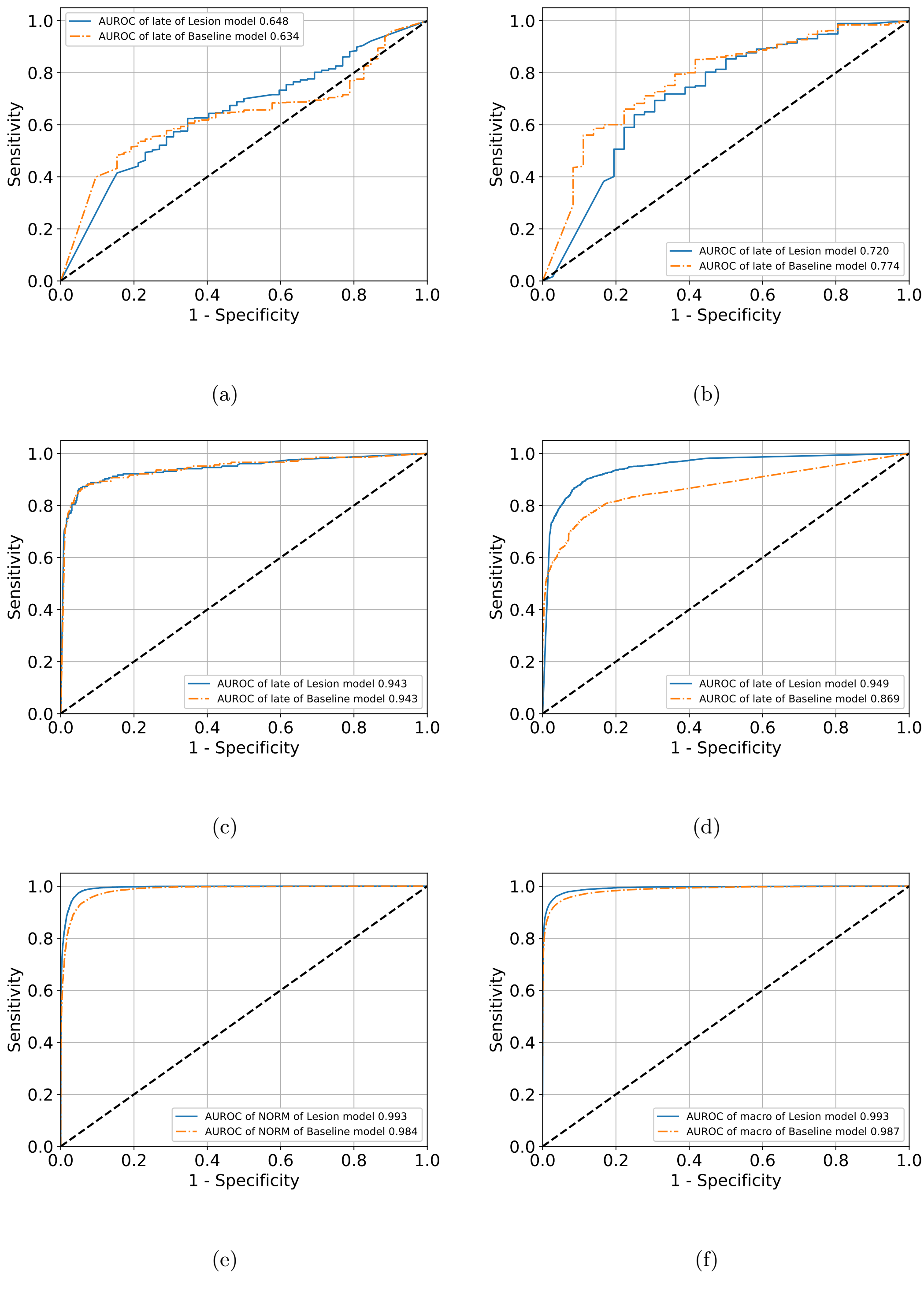

Figure S3: Comparison of ROC curves of Baseline model and Lesion-Focused model in the test set
