# Supplementary material for: A deep-learning pipeline for the diagnosis and grading of common blinding ophthalmic diseases based on lesion-focused classification model
Source: Front Artif Intell. 2024 Sep 11;7:1444136. doi: 10.3389/frai.2024.1444136 (PMC11422385; doi:10.3389/frai.2024.1444136)
Supplement: Supplementary file 7 [file Data_Sheet_6.PDF]

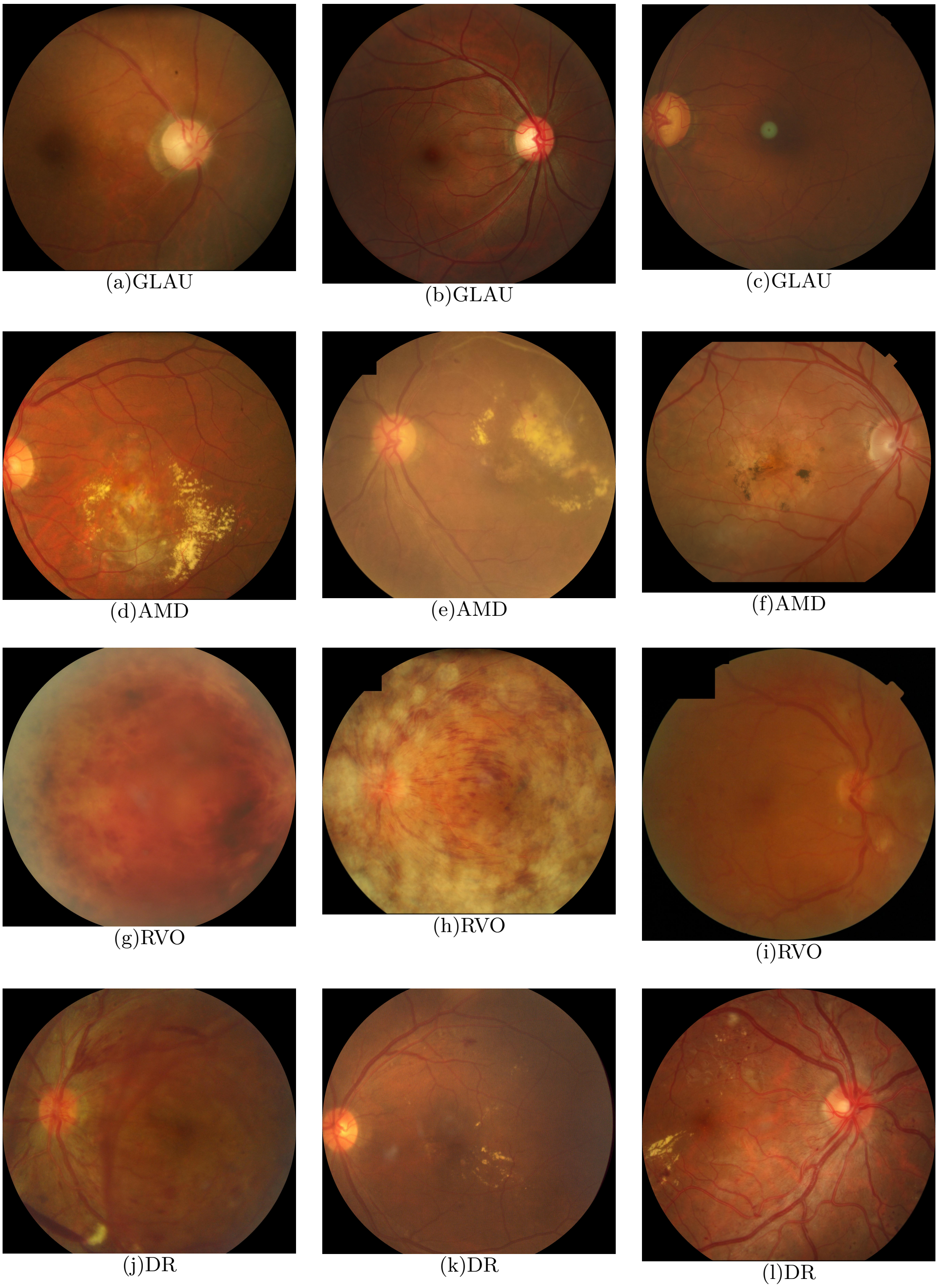

Figure S6: Three fundus images were randomly selected from the database for each disease. (a) ~(c) are Glaucommas, (d) ~(f) are AMDs, (g) ~(i) are RVOs, and (j) ~(l) are DRs.
