# Supplementary material for: A deep-learning pipeline for the diagnosis and grading of common blinding ophthalmic diseases based on lesion-focused classification model
Source: Front Artif Intell. 2024 Sep 11;7:1444136. doi: 10.3389/frai.2024.1444136 (PMC11422385; doi:10.3389/frai.2024.1444136)
Supplement: Supplementary file 8 [file Data_Sheet_7.PDF]

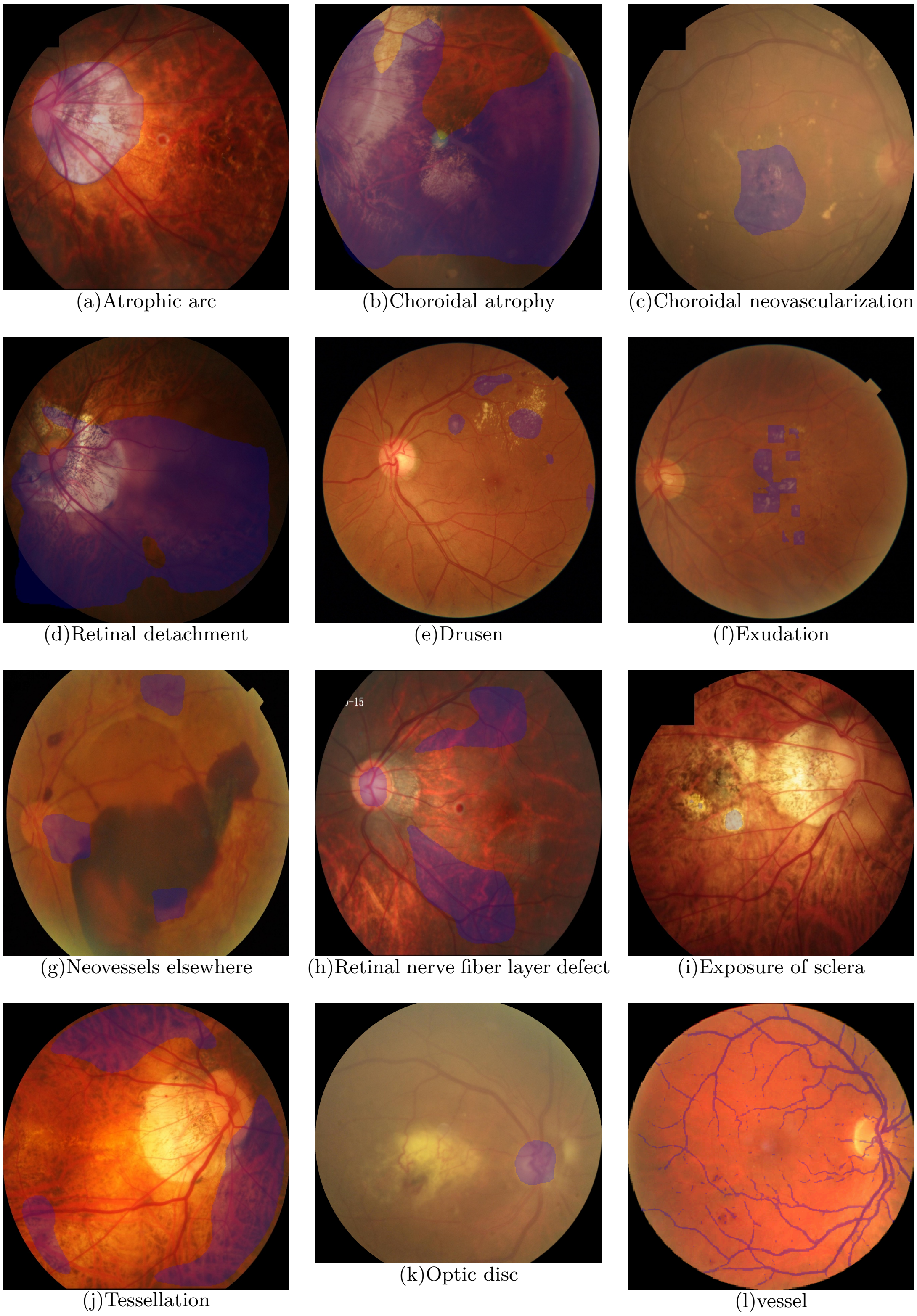

(a)Atrophic arc (b)Choroidal atrophy (c)Choroidal neovascularization

(d)Retinal detachment (e)Drusen (f)Exudation

(g)Neovessels elsewhere (h)Retinal nerve fiber layer defect (i)Exposure of sclera

(j)Tessellation (k)Optic disc (l)vessel

Figure S7: Semantic segmentation results of the fundus lesions. One image was randomly selected from the segmented image database for each lesion.
