# Supplementary material for: A deep-learning pipeline for the diagnosis and grading of common blinding ophthalmic diseases based on lesion-focused classification model
Source: Front Artif Intell. 2024 Sep 11;7:1444136. doi: 10.3389/frai.2024.1444136 (PMC11422385; doi:10.3389/frai.2024.1444136)
Supplement: Supplementary file 9 [file Data_Sheet_8.PDF]

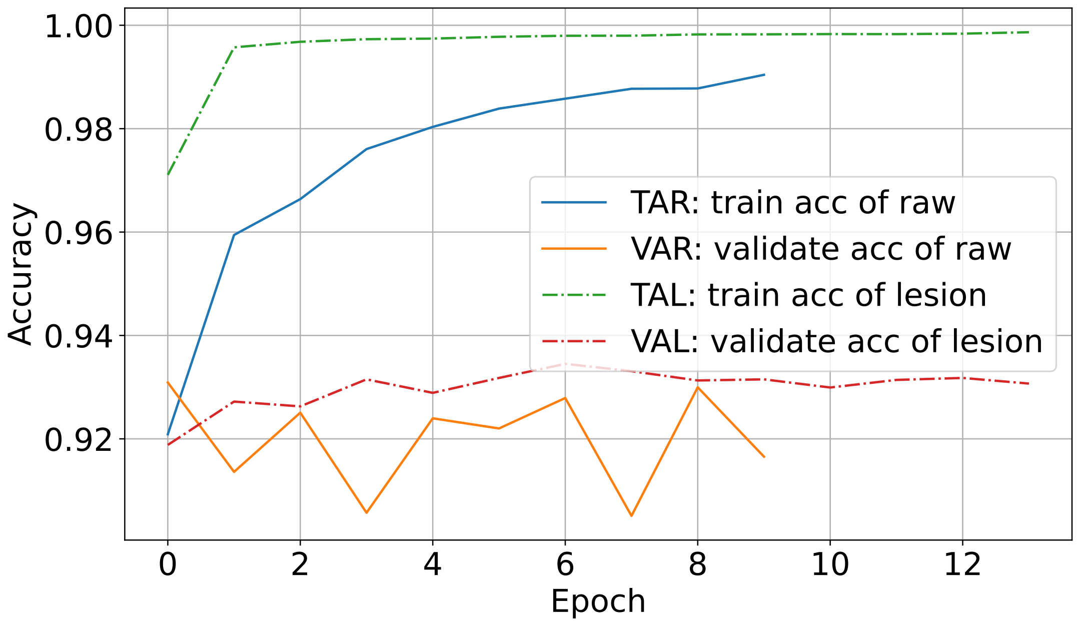

Figure S8: Illustrates a comparative analysis of the accuracy throughout the training process of two types of intelligent diagnosis algorithms: those using original fundus images and those employing lesion attention fundus images. In this context, TAR (Training Accuracy - Baseline) and VAR (Validation Accuracy - Baseline) denote the accuracy metrics for the training and validation sets of the algorithm using original fundus images. Conversely, TAL (Training Accuracy - Lesion-Focused) and VAL (Validation Accuracy - Lesion-Focused) represent the accuracy metrics for the training and validation sets of the algorithm that utilizes lesion attention fundus images. This comparison aims to evaluate the effectiveness of incorporating lesion attention in the analysis of fundus images for disease diagnosis.
